# Outcomes of Free Vascularized Fibular Grafts in Treating Massive Forearm Skeletal Defects

**DOI:** 10.3390/jpm14090973

**Published:** 2024-09-14

**Authors:** Panagiotis Konstantinou, Lazaros Kostretzis, Athina Zacharoula Ditsiou, Ioannis Samaras, Pericles Papadopoulos, Konstantinos Ditsios

**Affiliations:** 1 2nd Orthopaedic Department of Aristotle, University of Thessaloniki, “G Gennimatas” Hospital, 54124 Thessaloniki, Greece; lazaros.kostrezis@gmail.com (L.K.); gsammd@hotmail.com (I.S.); perpap@otenet.gr (P.P.); ditsiosk@otenet.gr (K.D.); 2 University Hospitals Birmingham NHS Foundation Trust, Birmingham B9 5SS, UK; 3 Healthcare Management, School of Social Science, Hellenic Open University, 26331 Patra, Greece; 4 Faculty of Health Sciences, School of Medicine, Aristotle University of Thessaloniki, 54124 Thessaloniki, Greece; ditsiouathina@gmail.com

**Keywords:** FVFG, large skeletal defect, upper limb, trauma

## Abstract

Introduction: Reconstructing long bone defects in the upper limbs, particularly the radius and ulna, poses significant challenges. These defects, resulting from trauma, tumors, infections, or congenital anomalies, require precise surgical intervention for functional restoration. Traditional non-vascularized autogenous bone grafts have limitations, such as resorption and limited biological activity. To address these challenges, free vascularized fibular grafts (FVFGs) have been developed, offering enhanced recovery by supplying nutrients and structural support, particularly in large defects or compromised vascularity. Materials and Methods: This retrospective study reviewed patients with significant forearm skeletal defects treated with FVFGs at our institution from January 2008 to January 2019. Included were patients with radius or ulna defects exceeding 8 cm due to trauma, tumor excision, or non-union fractures. Data on demographics, clinical details, surgical techniques, and outcomes—including graft union time, complications, range of motion, and the disabilities of the arm, shoulder and hand (DASH) scores—were analyzed. Results: Eight patients, with a mean age of 27.6 years and an average defect length of 9.8 cm, were included. All patients achieved graft union within an average of 4 months, with no tumor recurrence or significant complications. Functional outcomes showed mean forearm pronation of 56.9 degrees, supination of 52.5 degrees, and a mean DASH score of 17.7. Conclusions: FVFG is a safe and effective technique for managing complex forearm bone defects, providing high union rates and good functional outcomes. It should be considered a primary option for large forearm skeletal defects.

## 1. Introduction

The reconstruction of upper extremities long bone defects, particularly involving the radius or the ulna, presents a significant challenge for orthopedic surgeons. These defects can result from trauma, tumors, infections, or congenital anomalies, and require precise surgical intervention to restore function [1,2]. The size of the defect significantly influences the choice of surgical approach. Based on the extension of the Orthopaedic Trauma Association Open Fracture Classification, bone defects are classified in three categories (D1, D2, and D3). Critical-size defects (D3) are further divided in three further subcategories: moderate (2 to <4 cm), major (4 to <8 cm), and massive (equal to and above 8 cm) [3]. While smaller bone defects are usually addressed with bone grafts, larger defects often necessitate more intricate reconstruction methods. The complexity increases further when there are additional complications, such as infections or issues with soft tissue coverage. Bone-repair techniques and materials, including bone grafts and their substitutes [4], have been used since 1911 [5] to address these defects, but non-vascularized autogenous bone grafts, despite their long history, have notable disadvantages such as resorption and limited biological activity [6]. To address these disadvantages Taylor et al. introduced in 1975 free vascularized fibular grafts (FVFGs) for post-traumatic upper limb reconstruction [7] Compared to other methods, such as non-vascularized bone grafts, external fixators, the induced membrane technique (Masquelet), and limb shortening, FVFG facilitates better functional recovery by supplying nutrients to the deep structures of the graft [8]. Furthermore, vascularized bone grafts deliver signals that promote bone formation, contribute osteoprogenitor cells, and offer a structural framework that facilitates new bone growth [9]. Additionally, its length, straight diaphysis, and minimal donor site morbidity make the fibula an ideal choice for long-bone reconstruction, particularly in cases involving extensive defects or compromised vasculature [10].

More complex reconstructions are also achievable with FVFG by transferring the fibula along with a cutaneous paddle, muscle tissue, or both [11]. This flexibility allows for the reconstruction of not only bone defects but also the surrounding soft tissues, providing a more comprehensive approach to complex injuries. Vascularized bone grafts, particularly the free vascularized fibula graft (FVFG), have proven highly beneficial for treating bone and osteocutaneous defects. However, the most frequent complication associated with using the free fibula flap for upper extremity bone defects is graft fracture [12]. However, despite its versatility and success in addressing challenging bone defects, there is a limited amount of literature focused on the long-term outcomes of FVFG in forearm bone reconstruction, highlighting the need for further research in this area.

The aim of this study was to assess the efficacy, safety, functional outcomes, and patient-reported outcome measures of the use of FVFG in the reconstruction of large skeletal forearm defects.

## 2. Patients and Methods

### 2.1. Study Population

**Scheme 1:**
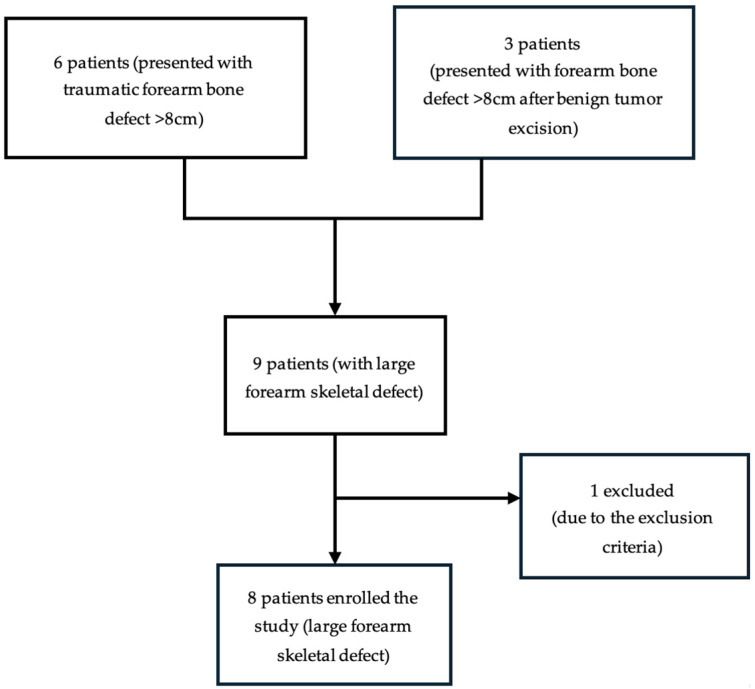
Patients enrolled in the study.

Our study included retrospectively all patients with significant skeletal forearm defects treated at our department between January 2008 and January 2019 using free vascularized fibular grafts (FVFGs). All procedures were performed by the same surgeon (K.D.) at the same institution. Inclusion criteria were: (1) patients with radius or ulna skeletal defects exceeding 8 cm due to severe bone trauma (massive bone defect), benign tumor excision, or fracture non-union resulting in non-fixable bone defects and (2) patients who agreed to participate in the study. Exclusion criteria were: (1) patients with metabolic disorders or diabetes mellitus, (2) patients with cardiovascular or peripheral artery disease, (3) patients with metastases in cases of tumor excision, (4) patients treated with a different method, and (5) patients with sustained injury or fracture on the same side during the follow-up period. Finally, nine patients presented to our department during this time period with a forearm bone defect greater than 8 cm. One patient was treated using the Ilizarov method with bone transport and was therefore excluded from our sample. The remaining eight patients were enrolled in this study, as shown in Scheme 1. All patients with forearm tumors underwent an MRI at the tumor site, as well as thoracic and abdominal CT scans, to rule out any possibility of metastasis. Ethical approval was obtained from the hospital ethics committee, and all patients completed an informed consent form to participate in the study. As this study is retrospective and was not anticipated at the time of treatment, all patients signed the relevant consent form during the latest follow-up.

### 2.2. Data Collection

Demographic data, including age and sex, as well as clinical details such as the cause of injury, previous operations, medical history, and the time from injury to final treatment, were obtained from the medical records of the patients. The follow-up process was conducted at upper limb clinics at 2 weeks post-op, 4 weeks, and subsequently on a monthly basis until radiologically confirmed bone union was achieved. A final clinical assessment, along with obtaining patient-reported outcome measures, was performed during a specific visit at the end of the follow-up period.

### 2.3. Surgical Technique

The entire procedure was performed under general anesthesia, supplemented with nerve block anesthesia of the upper limb to primarily manage post-operative pain. The patient was positioned supine on the surgical table, with a sand or liquid bag positioned under the buttock of the limb designated as the fibular donor site to maintain a slight internal rotation. Optimal coordination involved two distinct surgical teams: one responsible for tumor excision or preparation of the forearm non-union site, graft implantation, fixation, and vessel anastomoses and another tasked with receiving and preparing the vascularized fibular graft. It is crucial to preserve the periosteal blood supply. To avoid compromising it, a 2 mm layer of muscle should be retained, giving the graft a characteristic marble-like appearance. Once the peroneal vessel pedicle is identified both proximally and distally, it is protected with malleable retractors before performing the osteotomy. After ensuring vessel protection, the surgeon rechecks the distal and proximal osteotomy sites to confirm that at least 10 cm of both the distal and proximal fibula will remain post-osteotomy. The osteotomy is then performed with an oscillating saw, with the site constantly irrigated with cold saline to prevent thermal osteonecrosis. The distal bone cut is made first, followed by the proximal cut, taking care to protect the superficial peroneal nerve during the latter. Following the osteotomies, the peroneal vessels are ligated distally, typically with hem clips, and the pedicle is dissected free from the surrounding muscles. As the procedure progresses proximally, it is important to ensure a pedicle length of 5 cm before ligating and cutting the pedicle.

**Figure 1:**
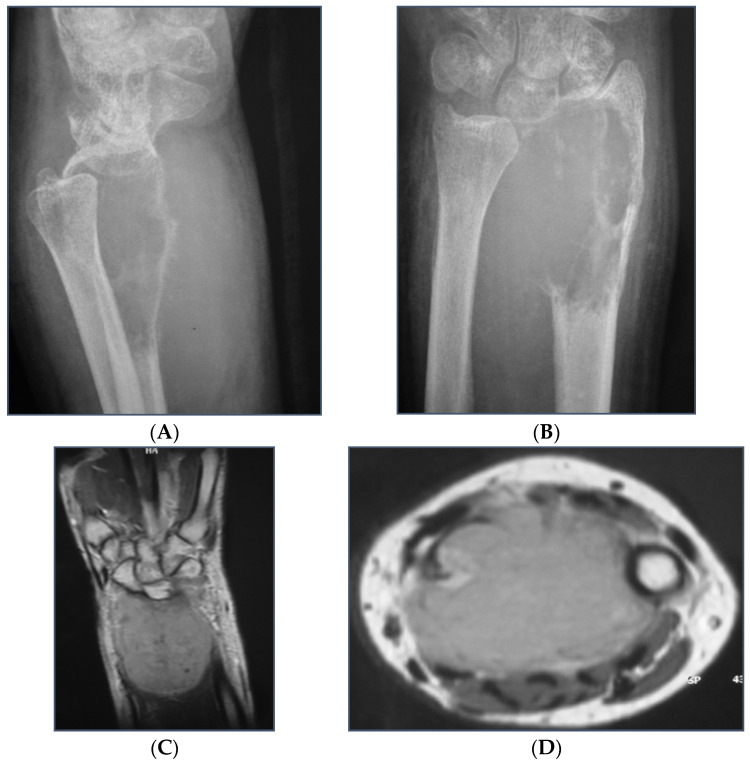

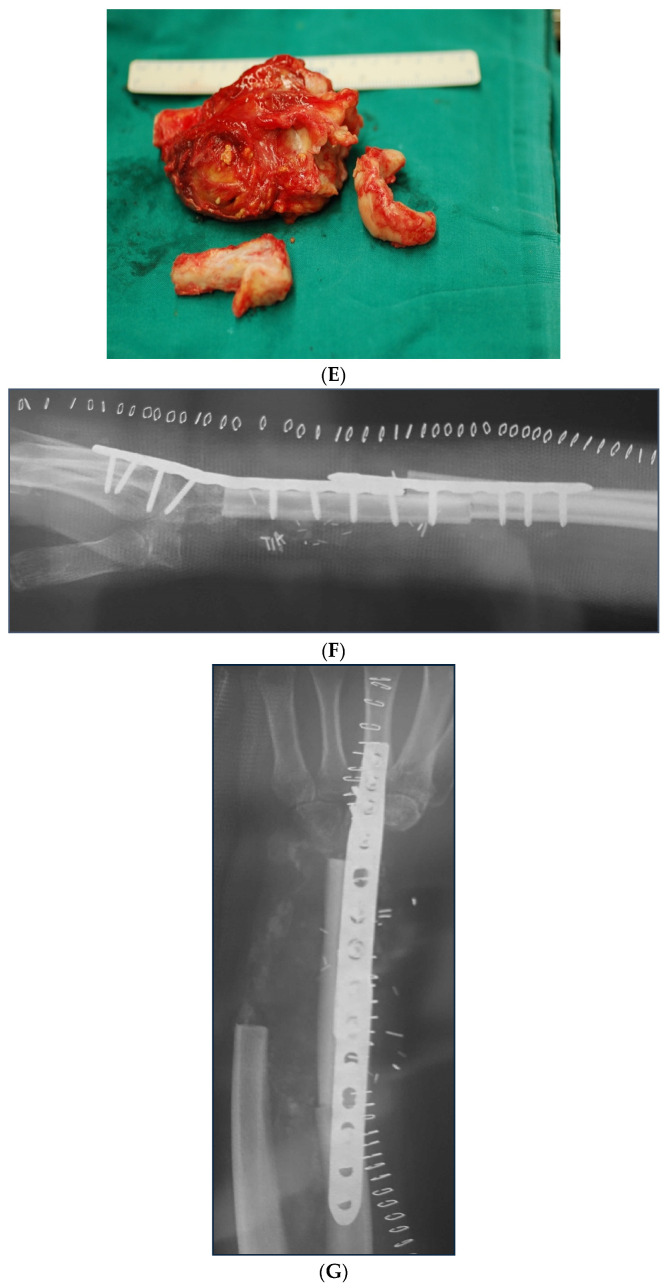
(**A**–**E**): A giant cell tumor of the distal radius that invades the distal articular surface, distal radioulnar joint, and palmar cortex of the radius. The selected treatment involves excision of the distal radius, including the pronator quadratus muscle, the proximal carpal row, and the distal third of the ulna. (**F**,**G**): Wrist arthrodesis using an intercalated free pedicled vascularized fibular graft was the treatment of choice.

**Figure 2:**
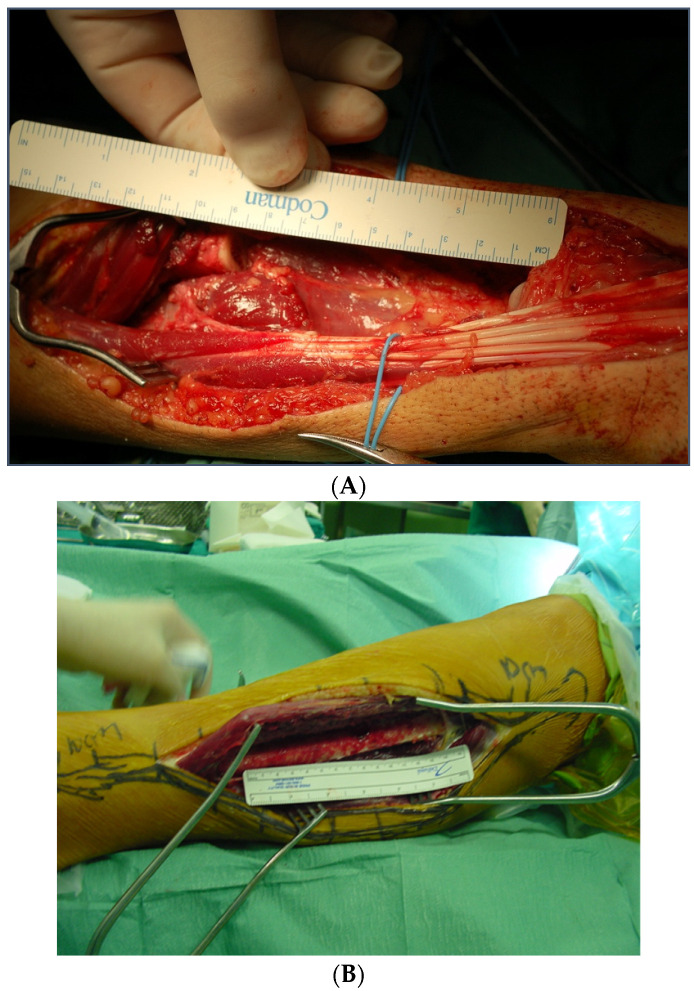
(**A**,**B**): Measuring the skeletal defect is essential to harvest the appropriate length of the fibular graft.

**Figure 3:**
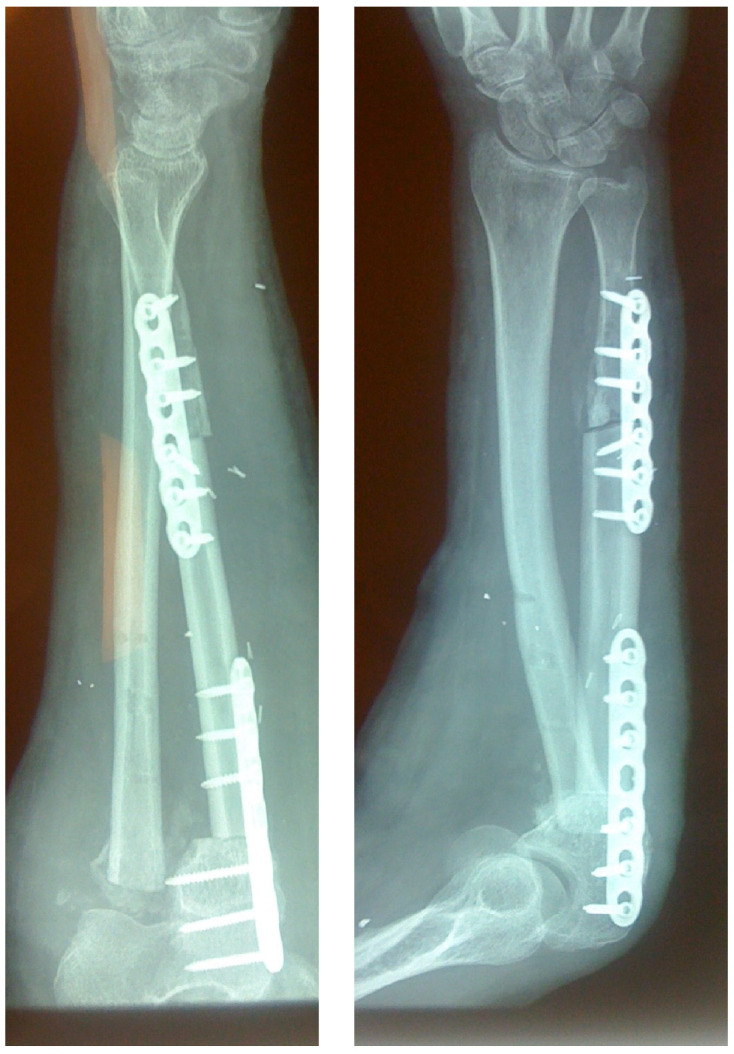
Large ulnar skeletal defect treated with FVFG, fixated using two LC-DCP plates.

In cases of bone fracture non-union, the surgeon excised scar tissue along with the sequestrum until reaching bleeding bone, following the removal of previously inserted metalwork. For tumor excision cases, the surgeon resected the bone tumor with clear margins or the entire compartment in cases of tumor-induced bone cortex breakage (Figure 1). At this stage, the length of the bone defect was measured to determine the appropriate length of the fibular graft to be harvested (Figure 2). The fibular graft fixation was accomplished using dynamic compression plates (DCPs) or anatomical plates, with a minimum of six cortices engaged on each side using either simple or locking screws (Figure 3). Once the osteosynthesis is completed, it is time to proceed with the vessel anastomosis. First, the peroneal vein is anastomosed to the radial or ulnar vein using 8-0 nylon monofilament sutures. Following this, the surgeon performs the arterial anastomosis using the same type of suture as previously. Simple interrupted stitches are preferred over a continuous suture technique and the anastomosis could be either end to end or end to side using a microscope.

### 2.4. Post-Operative Treatment

We implemented a backslab splint for 2–3 weeks to manage pain and support soft tissue healing. Patients were encouraged to engage in active contraction exercises for the digits, elbow, and shoulder muscles starting the day after the operation. The splint was removed at the time of stitch removal, allowing for forearm mobilization. Since stable osteosynthesis had been achieved, passive mobility was permitted at this stage, followed by active physiotherapy six weeks after the operation. Loading on the limb was advised to be avoided until radiographic evidence of graft union was present. Loading on the donor site was allowed based on the patient’s tolerance.

### 2.5. Study Outcomes

The measured outcomes of this study included: the time to graft union, the presence of complications (recipient or donor site) or reoperations, local recurrence in case of tumor excision, forearm range of motion (pronation and supination), wrist range of motion (flexion and extension), the DASH score [13], and recurrence (regional or remote) for cases of bone tumor. Graft union was assessed using simple X-rays during patient follow-up, noting the time when union was confirmed according to the X-ray report. Range of motion (ROM) and DASH score were recorded during a face-to-face examination at the end of the follow-up for each case. The DASH score had previously been translated and validated for the Greek population in a prior study [14]. Data are presented with means, along with standard deviations (SDs) and ranges for the continuous variables.

## 3. Results

### 3.1. Demographics

Eight patients participated in this study, comprising five males and three females, with a mean ± SD age of 27.6 ± 7 years (range, 14–38 years). Among them, five patients had traumatic bone defects, while three had defects resulting from bone tumor resection. Six cases involved defects in the radius, while the remaining two cases involved defects in the ulna. The three tumor cases were diagnosed as giant cell tumors with no evidence of remote metastasis. In one case, there was bone cortex breakage, leading to a procedure involving excision of the entire compartment, along with first-row carpectomy and wrist arthrodesis using FVFG. The remaining five cases involved severe comminuted fractures. Four of these cases (three in the radius and one in the ulna) were closed fractures initially treated with ORIF plate fixation with or without bone grafting, while the last case was an open ulnar fracture stabilized initially with external fixation. The mean time from initial injury to FVFG was 9.6 ± 5.9 months (range, 1–17). In cases involving bone tumors, there were no prior operations except for biopsy. The mean ± SD length of bone defect was 9.8 ± 1.3 cm (range, 8–12 cm). No patient was lost to follow-up. The mean follow-up time was 84.5 ± 14.7 months (range, 64–110 months).

### 3.2. Time to Union—Complications

All patients achieved graft union at a mean time of 4 ± 0.7 months (range, 3–5 months). During the follow-up period, no complications were recorded at either the recipient or donor sites, and no additional reoperations were required for the definitive treatment of the patients. There were no instances of regional or remote tumor recurrence identified. 

### 3.3. Functional Outcomes (ROM—DASH Score)

At the mean post-op follow-up time, the mean forearm pronation was 56.9 ± 20.1 degrees (range, 30–85 degrees) and supination was 52.5 ± 17.5 degrees (range, 20–80 degrees). Wrist flexion averaged 33.1 ± 17.3 degrees (range, 0–60 degrees), while wrist extension averaged 23.1 ± 11.6 degrees (range, 0–40 degrees). The mean DASH score at that time was 17.7 ± 12 (range, 7.5–45). All the aforementioned data are presented in detail in Table 1.

## 4. Discussion 

The treatment of forearm skeletal defects is a challenging procedure that often requires complex operations performed in one or more stages. Over the years, various methods have been employed for treating forearm skeletal defects. In 2002, Davey and Simonis [15] introduced a modification of the Nicoll bone grafting technique for treating forearm non-union of the radius and/or ulna in 19 patients. Their approach involved using corticocancellous bone from the iliac crest and plate fixation with compression. Additionally, the Masquelet technique has been utilized for bone defects in the hand and wrist [16], as well as for defects involving one of the two bones in the forearm [17]. In 2015, a case report was published describing the successful use of this technique for treating skeletal defects [18] in both forearm bones. The single-bone forearm technique was described in 1975 as a reconstructive surgical technique to address large skeletal defects following severe trauma, infection, or tumor excision [19]. Another option for segmental skeletal defects of the forearm could be distraction osteogenesis through bone transportation using external fixation devices, as described by Ditsios et al. [20]. They treated a radius osseous defect of 8 cm and a simultaneous ulnar defect of 4 cm in the same forearm using this method. The usage of bone allografts has been applied in a large segmental defect in the forearm after tumor excision [21] as well as in cases of infected forearm non-unions [22]. 

The free vascularized fibula graft technique was first described in 1975 by Taylor et al. [7]. Since then, it has become the gold standard vascularized graft in long bone pathologies for defects larger than 6 cm due to trauma, tumors, osteomyelitis, or non-union [23,24]. One reason is that the fibula’s dual endosteal and periosteal circulation enables osteotomies to be performed without compromising the bone’s vascularization. This fact allows this graft to be used even in cases with poor vascularization resulting from severe trauma or infection. With this technique, it is possible to reconstruct one or both forearm bones in a single operation, even if there is a soft tissue defect, as FVFG can be utilized as an osseocutaneous graft as well [25]. This technique could also be combined as a chimeric flap, along with a sural neurocutaneous flap, for the reconstruction of complex tissue defects in forearms with segmental bone and nerve defects [26]

In our case series, the mean time needed for consolidation was 4 ± 0.7 months (range, 3–4.5 months), which is considered comparable to previous findings [25,27,28]. Slightly prolonged consolidation mean times are reported for post-tumor-resection humeral cases (7 months) and forearm cases (6.5 months) by Shahzad et al. [29]. Claxton et al. increase this duration to 9 months post-operation for primary union in oncologic upper extremity cases [30]. We prefer stable fixation of the fibular graft with two plates at both ends of the graft because we believe that the stability provided by this technique promotes better and faster bone healing compared to elastic nails. Our consolidation rate was 100% as graft union was achieved in all of our cases. Similar rates were reported by Dell P.C. et al. [31] and Malizos, K.N. et al. [32]. A recent study also reported a union rate of 90% in ten cases of upper limb traumatic skeletal defects using FVFG. However, non-union was noted in a humeral case, raising the consolidation rate in forearm cases to 100% [33]. Even in studies involving non-union complications, the rate of free vascularized fibular graft union is high according to the literature, with union rates ranging between 88.9% and 94% [27,34,35,36]. On the other hand, Claxton et al. recently reported a lower rate of primary union in 20 out of 28 patients treated with FVFG for upper limb skeletal defects resulting from tumor excision. In the same study, female gender was positively associated with failure of primary union [30]. Shahzad et al. reported nearly identical results regarding consolidation of post-tumor-excision skeletal defects in the humerus using FVFG (79%), with the consolidation rate rising to 100% for forearm cases in the same study [29]. The consolidation rate of FVFG, whether osteocutaneous or not, remains high even in cases with different etiologies (bone defects, pseudoarthrosis, gunshot wounds, giant cell tumors) in both upper and lower extremities, supporting that FVFG can ensure acceptable results even in demanding cases [37]. In our study, no complications were noted from the donor site, and there were no other complications from the recipient site, such as infection, graft resorption, or tumor recurrence during patient follow-up. In the literature, apart from non-union, other complications that have been reported include graft osteitis [25], artery thrombosis [28,29], venous thrombosis of the graft [27], wound dehiscence [33], proximal radioulnar synostis [33], local infection [37], venous thrombosis of the graft, and hematoma [38].

The functional result, in terms of range of motion (pronation, supination, wrist flexion, and wrist extension), is comparable to that of other studies in the literature [27,28]. The mean DASH score in our study was found to be 17.7 ± 12 (range, 7.5–45), which is considered to be equal to the ones reported in Cano-Luis et al.’s study (average 17.1) [25]. In this study, the DASH results were measured at the end of follow-up, which averaged 13.9 months, significantly different from our evaluation of the DASH score, which was conducted at a mean time of 84.5 months post-op. Even better functional results, measured by the DASH score, are reported by Jussi Petteri Repo et al., with a mean DASH score in the group of adults who had undergone radial (*n* = 2) or humeral (*n* = 1) reconstruction of 6.7 points (range, 0–53.5) [11]. Even for forearm oncologic cases, Shahzad et al. reported very good and excellent functional results, with sixteen patients experiencing no pain or restrictions in activities of daily living (ADLs), one patient experiencing occasional pain but no ADL restrictions, and two patients experiencing occasional pain and some ADL restrictions [29].

The relatively low number of patients and the heterogeneity of the cases in this study may have affected the final results and led to such outcomes. The lowest functional result, according to the DASH score in our study, was noted in a tumor resection case with wrist fusion as the final treatment, and this low result may be attributed to this type of operation and not graft choice. 

The limitations of this study include the relatively low number of patients, as these cases are not very common, making it difficult to achieve a relatively large sample size. Furthermore, our study lacks a control group for comparing the free vascularized fibular graft to other techniques through bivariate analysis, which limits our ability to establish the superiority of this method over available alternatives. Additionally, the inclusion of both traumatic and tumor cases introduces variability in the type and extent of skeletal defects, complicating direct comparisons between patients. This variability may affect outcomes differently depending on the underlying cause of the defect. Another limitation is that the DASH score was assessed only at the final follow-up rather than at consistent post-operative intervals, as seen in other studies. This could impact the comparability and interpretation of our results, potentially leading to either an overestimation or underestimation of functional recovery based on the level of rehabilitation achieved during the extended follow-up period. One strength of this study is that all operations were performed by the same surgeon, and the follow-up period lasted almost seven years, which is considered more than adequate time for trauma cases. Further comparative studies with longer follow-up are needed to evaluate all the available therapeutic techniques for such complicated cases.

Since the sample size in our study, as well as in previously published studies, is considered relatively small, and given the lack of published comparative studies on treatment methods for large forearm skeletal defects, it is crucial to personalize the treatment approach for each patient. Therefore, the treating physician must assess the size of the skeletal defect, as this affects the duration of treatment, particularly when bone transport with a frame is selected. Additionally, the patient’s medical history and consent for having an external fixator on the forearm for an extended period must be considered, including the risk of pin track infection due to prolonged frame application. The Masquelet technique requires multiple operations to achieve final treatment, increasing the risk of complications and additional surgeries. Bone grafting (autologous or allogenous) could be a viable option if the soft tissue envelope is well preserved, which is not always the case in trauma scenarios. The FVFG, while demanding, offers the advantage of addressing the issue in a single theater session. It provides biological support for severe soft tissue damage and serves as a safe alternative in cases of infection. The promising bone tissue engineering technology may provide the medical team and patients with a 3D bioprinted custom living and vascularized bone graft [39]. This technology allows for direct application to the patient, ensuring perfect adjustment based on the patient’s anatomy and eliminating donor site comorbidities. Moreover, this promising technology offers the advantage of creating personalized, vascularized scaffolds. However, despite the potential of 3D bioprinting to produce tailored, living bone transplants with integrated vasculature, it has yet to be explored in clinical applications. Several hurdles remain, such as preserving cell viability, achieving sufficient cell density, ensuring precise spatial differentiation within the 3D structure, and establishing connections with the host vasculature. Addressing these challenges will be critical for future research in this field.

In summary, the surgeon must evaluate the patient’s medical history, the size of the skeletal defect, the condition of the soft tissues, the patient’s preference regarding treatment methods, and the potential for existing or future infections to make an appropriate, personalized decision. 

## 5. Conclusions

According to our findings, we consider the use of a free vascularized fibular graft as a safe and reliable option for treating large skeletal defects of the forearm caused by severe trauma, or tumor. This technique yields a high rate of union along with very good functional outcomes, even in cases complicated by severe soft tissue trauma or tumor. This graft combines anatomical and biomechanical characteristics, which, when coupled with the option to use it alongside a musculocutaneous flap, appears to be an exceptional choice for comprehensive forearm reconstruction in a single operation.

## Figures and Tables

**Table 1:** Patients’ characteristics and clinical details.

Case	Sex	Age (Years)	Type of Injury (Diagnosis)	Site of Bone Defect	Initial Treatment (Previous Operation)	Time from Injury to FVFG (Months)	Graft Length (cm)	Bone Union (Months)	Forearm ROM (Degrees)		Wrist ROM (Degrees)		DASH Score	Follow-Up (Months)
									Pronation	Supination	Flexion	Extension		
1	F	14	Giant cell tumor	R	-	-	8	3	85	80	60	30	7.5	98
2	M	27	Closed fracture	R	ORIF + bone graft	12	10	4	70	60	40	20	12.5	74
3	F	32	Closed fracture	U	ORIF	17	9	4.5	50	60	30	20	18.3	82
4	M	29	Closed fracture	R	ORIF + bone graft	8	12	4	50	60	25	30	17.5	78
5	M	38	Open fracture	U	External fixation	1	9.5	5	70	40	45	40	11.7	64
6	F	24	Closed fracture	R	ORIF	10	8.5	3	30	20	30	25	20.8	78
7	M	31	Giant cell tumor	R	-	-	11	4.5	30	50	0	0	45	110
8	M	26	Giant cell tumor	R	-	-	10.5	4	70	50	35	20	8.3	92

F = female; M = male; R = radius; U = ulna; ORIF = open reduction internal fixation.

## Data Availability

The datasets presented in this article are not readily available because privacy and ethical restrictions. Requests to access the datasets should be directed to corresponding author.
